# Harper’s Magazine for February

**Published:** 1890-03

**Authors:** 


### HARPER'S MAGAZINE, for February, contains a remarkable article from the pen of Mark Twain, entitled, "A Majestic Literary Fossil."

In this article the incomparable Mark rakes over the literature of the old school of medicine of a hundred years ago and more, and in his facetious way presents its absurdities in their most ludicrous light. For instance, he quotes from an old book of medicine that "the ashes of an ass' hoof mixed with woman's milk is good for chilblains." "The constant use of milk is bad for the teeth, causes them to rot, and loosens the gums."

Paracelsus saw a plaster that had such drawing quality that it could draw a man's lungs up into his mouth, causing him to be suffocated. It could draw enough water to fill a cistern, and was strong enough to tear branches from trees and to draw a cow up into the air. This must have been a fly-blister.

He quotes a wonderful elixir composed of "a great peck of garden snails," earth worms, rosemary, tumeric, bark of barberry trees, goose-dung, strong ale, and other efficacious remedies, suitably mixed together. Then having ventilated these follies in that inimitable manner which has made this author so famous, he closes with the following paragraph which we quote entire:

"When you reflect that your own father had to take such medicines as the above and that you would be taking them to-day yourself but for the introduction of Homœopathy, which forced the old-school doctor to stir around and learn something of a rational nature about his business, you may honestly feel grateful that Homœopathy survived the attempts of the allopathists to destroy it, even though you may never employ any physician but an allopathist while you live."

